# Dental caries in children and adolescents with juvenile idiopathic arthritis and controls: a multilevel analysis

**DOI:** 10.1186/s12903-021-01758-y

**Published:** 2021-08-25

**Authors:** Elisabeth G. Gil, Anne N. Åstrøm, Stein Atle Lie, Marite Rygg, Johannes Fischer, Annika Rosén, Athanasia Bletsa, Keijo Luukko, Xie-Qi Shi, Josefine Halbig, Paula Frid, Lena Cetrelli, Karin Tylleskär, Karen Rosendahl, Marit S. Skeie

**Affiliations:** 1 Department of Clinical Dentistry, The Faculty of Medicine, University of Bergen, Årstadveien 19, 5009 Bergen, Norway; 2 Department of Clinical and Molecular Medicine, Norwegian University of Science and Technology (NTNU), Trondheim, Norway; 3 Department of Pediatrics, St. Olav’s Hospital, Trondheim, Norway; 4 Department of Oral and Maxillofacial Surgery, Haukeland University Hospital, Bergen, Norway; 5 Oral Health Centre of Expertise in Western Norway-Vestland, Bergen, Norway; 6 Department of Oral Maxillofacial Radiology, Faculty of Odontology, Malmö University, Malmö, Sweden; 7 Public Dental Health Competence Centre of Northern Norway (TkNN), Tromsø, Norway; 8 Department of Clinical Dentistry, The Arctic University of Norway, Tromsø, Norway; 9 Department of Otorhinolaryngology, Division of Oral and Maxillofacial Surgery, University Hospital of North Norway, Tromsø, Norway; 10 Center for Oral Health Services and Research (TKMidt), Trondheim, Norway; 11 Department of Pediatrics, Haukeland University Hospital, Bergen, Norway; 12 Department of Radiology, University Hospital of North Norway, Tromsø, Norway

**Keywords:** Adolescent, Child, Dental care for children, Dental caries, Oral health, Juvenile idiopathic arthritis, Multilevel analyses

## Abstract

**Background:**

Optimal utilization of dental caries data is crucial in epidemiological research of individuals with juvenile idiopathic arthritis (JIA). The aims were to: explore whether caries is more prevalent among children and adolescents with JIA compared to controls; examine presence of caries according to JIA group, socio-behavioral and intraoral characteristics, and the extent to which surface-specific caries varies between and within individuals; assess whether surface-specific caries varies according to JIA group and dentition; and investigate whether disease-specific clinical features of JIA are associated with presence of caries.

**Methods:**

In this comparative cross-sectional study, calibrated dentists examined index teeth (primary 2. molars, 1. permanent molars) of 4–16-year-olds with JIA (n = 219) and matched controls (n = 224), using a detailed caries diagnosis system (including enamel caries). JIA-specific characteristics were assessed by pediatric rheumatologists and socio-behavioral information collected by questionnaires. Multilevel mixed-effect logistic regressions reporting odds ratios (OR) with 95% confidence interval (CI) were applied (caries at surface level as outcome variable). Potential confounders were adjusted for, and the effect of dependency of surface-specific caries data was estimated by calculating intra-class correlation coefficients (ICC).

**Results:**

At individual level, no significant difference in caries prevalence was found between individuals with JIA and controls, regardless of inclusion of enamel caries. Proportion of enamel lesions exceeded dentine lesions. JIA was not associated with presence of caries, but in both groups, low maternal educational level was associated with presence of caries (OR: 2.07, 95% CI: 1.24–3.46). Occlusal and mesial surfaces, compared to buccal surfaces, had generally higher OR according to presence of caries than distal and lingual surfaces (ICC = 0.56). Surface-specific caries in the permanent dentition differed significantly according to group affiliation. Some JIA disease-specific variables were suggested to associate with presence of caries.

**Conclusions:**

No overall difference in caries prevalence between individuals with JIA and controls was observed, but for both groups, low maternal educational level and tooth surface associated with presence of caries. Associations between JIA disease-specific variables and presence of caries cannot be excluded. Due to predominance of enamel lesions, the potential of preventative dental strategies is considerable.

**Supplementary Information:**

The online version contains supplementary material available at 10.1186/s12903-021-01758-y.

## Background

Juvenile idiopathic arthritis (JIA) is the most common chronic arthritis disease in children and adolescents and is an important cause of short and long-term disability [1]. JIA is not a single disease, but a term that encompasses all forms of arthritis of unknown etiology, starting before the age of 16 and persisting for at least 6 weeks [2]. The prevalence and incidence rates of JIA varies across populations. A prevalence of 32.6/100,000 children and an incidence rate of 8.3/100,000 children per year has been estimated for Caucasians [3]. High incidence rates in northern European countries have been reported, and in Northern and Central Norway, an incidence rate of 23/100,000 children per year have been demonstrated [4, 5].

A recent systematic review and meta-analysis by our research team focusing on caries and other oral health conditions among children and adolescents with JIA, found no significant difference in mean dmft/DMFT (decayed/missing/filled teeth) between the groups with and without JIA [6]. This finding contrasts previous reviews by Walton et al. [7] and Synodinos et al. [8], reporting a high prevalence of caries among children and adolescents with JIA. Improved medical and effective overall treatment of JIA [9, 10], especially in countries with well-established health care services, have presumably reduced some of the prevailing dental caries risk factors associated with this disease. Also, development of current alternative sweeteners and sugar alternatives in pediatric drugs in general [11] may have reduced the risk for future caries development and explains why caries prevalence in young individuals with JIA in recent years have become comparable with those of the general population. However, children and adolescents with JIA face additional challenges associated with caries development. For example salivary alterations and reduced salivary flow rate have been reported [12, 13], which may reduce the protective role of the saliva and lead to poor oral hygiene [13]. Additionally, antirheumatic medication such as different synthetic disease-modifying antirheumatic drugs (sDMARDs), especially the commonly used Methotrexate, may cause side-effects like nausea, vomiting and stomatitis [14, 15], presumably constituting a negative impact on oral health behaviours associated with oral hygiene and dietary habits.

Insufficient sample sizes [6] and non-optimal utilization of data [16] are common challenges in caries epidemiology concerning children and adolescents with JIA. The aggregated dmf/DMF index does not account for the hierarchical structure of the data with surfaces clustered within teeth and teeth clustered within individuals. Applying data on tooth and surface level require adjustment of the clustered nature of the data. Neglecting to adjust for clustering may result in smaller standard errors and increased likelihood of falsely rejecting the null hypothesis (Type I error) [17]. Hence, multilevel modelling in the analysis of surface-specific caries is needed.

There is a need for more high-quality studies with sufficient sample sizes and better exploitation of caries data, to fill the knowledge gaps concerning caries status among children and adolescents with JIA and to assess whether rheumatological characteristics are associated with caries. The aims of this study were to explore whether caries is more prevalent among children and adolescents with JIA compared to matched controls. Independent of JIA group, we also examined the presence of caries according to socio-behavioral and intraoral (within mouth) characteristics and the extent to which surface-specific caries varies between and within individuals. Furthermore, we assessed whether surface-specific caries varies according to JIA group and dentition (primary/permanent molars). Finally, and specifically for patients with JIA, we investigated whether clinical features such as JIA category, age at onset of JIA, disease duration, use of medication, or remission status were associated with the presence of caries.

## Methods

### Study design

This comparative cross-sectional study is based on baseline data from NorJIA,^1^ a prospective longitudinal multicenter study performed in the period 2015–2020 and registered at ClinicalTrials.gov (No: NCT03904459). Inclusion criteria were children or adolescents (4–16 years old) diagnosed with JIA by a pediatric rheumatologist according to the criteria by the International League of Associations for Rheumatology (ILAR) [2]. Exclusion criterion was lack of written informed consent. There were no participants with major medical comorbidities such as congenital facial anomalies, skeletal dysplasia, or malignancies in the study population. The medical and caries data for the present sub-study were collected between April 2015 and August 2018. To validate acceptable entry of data, 10% of the data input was rechecked.

### Sample size calculation

The sample size calculation is presented in Additional file 1.

### Participants

**Fig. 1:**
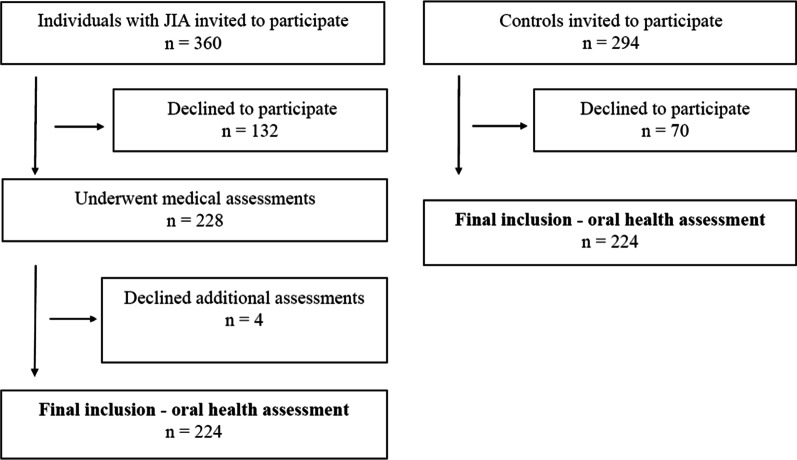
Participant flow diagram of children and adolescents with juvenile idiopathic arthritis (JIA) and controls

A total of 360 individuals with JIA were invited to participate, 228 underwent a medical examination, and a total of 224 were included in the final oral health examination (Fig. 1). The participants were recruited from three university hospitals located in western, central, or northern Norway. The participants with JIA were matched 1:1 with a control group according to sex, age, center site, and mothers’ country of origin (western or non-western origin). The controls were recruited from seven Public Dental Service (PDS) clinics and did not have JIA and substantially no other chronic diseases (Additional file 2, Table S1). The participants with JIA and their matched controls originated from the same geographical areas (western, central, or northern Norway). The PDS clinics were localized in both rural and urban areas. The controls had their appointments combined with planned regular oral health checks and received two cinema tickets as incentives for participation. The oral health examination was comprehensive, but subsequently this sub-study was restricted to only cover caries disease.

### Questionnaires and construction of variables

All participants (and/or caregivers, as appropriate) were asked to complete a questionnaire concerning sociodemographic and behavioral characteristics. The questionnaire version given to participants under the age of 12 years was slightly different than the version given to those 12 years of age and older. For all participants, the questionnaire included an item regarding their mother’s country of origin. If this item was not filled in, it was decided to identify the mother’s background-origin by the family name of the participant (in Norway it is fairly common for the family name of a child to include both the father’s and mother’s name). Other variables obtained from the questionnaire were educational level of caregivers, number of caregivers in the household, frequency of toothbrushing, frequency of tooth flossing during the last 3 months, gingival bleeding during toothbrushing, and pain or discomfort during toothbrushing. Additional questions given to participants under the age of 12 years were age of starting toothbrushing, whether they get assistance if tooth flossing is performed, whether they had cordial/milk from a bottle after the age of 1 year, and whether drinks or food were offered or available in bed in the evening/during the night. The coding is presented in Additional file 3, Table S1A and Table S1B. Lower and upper jaws were categorized as (0) mandible and (1) maxilla. The oral cavity was dichotomized into (0) right side (first and fourth quadrant) and (1) left side (second and third quadrant). The dentition was dichotomized into (0) primary second molars and (1) permanent first molars. Tooth surfaces were categorized as (0) buccal surface, (1) distal surface, (2) lingual surface, (3) mesial surface and (4) occlusal surface.

The JIA-specific clinical variables included were JIA category, medication, age of disease onset in years, disease duration in years, activity/remission status, physician’s global assessment of disease activity visual analogue scale (MDgloVAS), patient/parent-reported global assessment of overall well-being visual analogue scale (PRgloVAS), Childhood Health Assessment Questionnaire (CHAQ) hygiene item tooth brushing. The coding is presented in Additional file 3, Table S1C.

### Examinations

Experienced pediatric rheumatologists at each of the three university hospitals were responsible for the examinations of the participants with JIA. Elaboration of the JIA-specific clinical background variables, collected by the pediatric rheumatologists, are presented in Additional file 4. Both children and adolescents with JIA and the controls underwent caries assessment based on bitewing radiographs (BW) combined with visual inspection as part of a thorough oral examination. Five dentists underwent calibration courses and exercises. The caries calibration sessions are described in Additional file 5. Decayed, missing, and filled surfaces were registered according to a detailed 5-graded diagnostic tool [18]. Grades 1–2 denoted enamel lesions and grades 3–5 dentin lesions. Missing teeth were defined as teeth extracted due to caries or indicated for extraction. From the age of 5 years BW were taken when there was intermolar contact, but not if fixed orthodontic appliances were present. If fixed orthodontic appliances were present, only occlusal surfaces were examined by visual inspection. To achieve maximum comparability of dentition in children at various shedding/eruption stages, only molars were assessed (at younger than 10 years, primary second molars, and at 10 years and older, permanent first molars). Norwegian studies have found primary second molars to be the teeth most susceptible to caries in 5-year-olds [19], and the first permanent molars the teeth mostly affected at 12–18 years of age [20].

### Statistical methods

Data were analyzed using SPSS version 25.0 (IBM Corp. Released 2013, IBM SPSS Statistics for Windows, Armonk NY: IBM Corp) and STATA version 16 (Stata Corp LP, College Station, TX). For reliability statistics, linear weighted Cohen’s kappa and percent agreement values were used. Mean and SD were used to describe continuous clinical and demographic variables. Chi-squared tests were used to assess differences in categorical variables between individuals with JIA and the control group. Because the data for presence of caries (0 = no, 1 = yes) had a clustered 3-level hierarchical structure with surfaces (level 1) clustered within teeth (level 2), and teeth clustered within individuals (level 3), random intercept logistic models (RIM) were fitted using multilevel mixed-effects logistic regressions. Illustration of the levels in the multilevel models, according to the dichotomous caries outcome variable and background variables are presented in Additional file 6, Table S1. The formulas estimated (using restricted maximum likelihood REML) using mixed effects (random intercept) logistic regression were:

$$ logit\left( {P\left( {Y_{ijk} = 1} \right)} \right) = \beta_{0} + \beta_{1}^{T} X_{ijk} + \beta_{0i} + \beta_{0ij} + e_{ijk} $$

$$ Y_{ijk} \sim Binomial\left( {n_{i} ,p_{i} } \right) $$

$$ \beta_{0i} \sim N\left( {0,\sigma_{v}^{2} } \right) $$

$$ \beta_{0ij} \sim N\left( {0,\sigma_{u}^{2} } \right) $$

$$ icc_{v} = \frac{{\sigma_{v}^{2} + \sigma_{u}^{2} }}{{\sigma_{v}^{2} + \sigma_{u}^{2} + \pi^{2} /3}} $$

$$ icc_{u} = \frac{{\sigma_{u}^{2} }}{{\sigma_{v}^{2} + \sigma_{u}^{2} + \pi^{2} /3}} $$

The multilevel models account for clustering of caries data for sites (k-level) within teeth (j-level) and within individuals (i-level). Intra-class correlation coefficients (ICC) for the matching pairs were tested using separate mixed effects logistic models. Sociodemographic and behavioral characteristics that were statistically significantly associated with JIA status and presence of caries (d_1-5_fs/D_1-5_FS > 0) were included as potential confounders in the mixed-effects logistic regression analysis. Additionally, clinical features statistically associated with presence of caries (d_1-5_fs/D_1-5_FS > 0) in unadjusted analysis and the main exposure variable (JIA/control group) were adjusted for in the mixed-effect logistic regression analysis. The effect of dependency of caries data at individual level and tooth level was assessed by calculating ICCs, applying the described formulas. The ICC will also express variations between teeth and individuals as a proportion of the total variance. ICC varies between 0 (implying caries is independent within individuals) and 1 (indicating no variation of caries within an individual). Post-hoc test was applied by Scheffe’s method to adjust significance levels in multiple comparisons in the multilevel mixed-effects logistics regressions. *p* values less than 0.05 were considered statistically significant.

### Ethical approval

The study was approved by the regional ethics committee (2012/542/REC). Approval was also received by leaders of different County Dental Health Authorities, at different Oral Health Centre of Expertise, and at the three pediatric departments at university hospitals. Written informed consent was signed before participation. This sub-study as part of the NorJIA study was registered at ClinicalTrials.gov (No: NCT03904459).

## Results

### Sample characteristics

Of the 360 eligible children and adolescents with JIA, 228 underwent the medical assessment, yielding a response rate of 63.3% (Fig. 1). The corresponding response rate for controls was 224/294 (76.2%). Mean age of participants with JIA and controls was 12.0 years (SD 3.2) (p = 0.98). In comparison, the mean age of the 132 eligible JIA patients who declined to participate was 10.5 (SD 3.5) years (*p* < 0.01). The proportion of girls was slightly higher for the participants with JIA, compared to the group of JIA patients who declined participation (59.2% vs 58.3%, *p* = 0.03).

After the medical examination, a further 4 declined participation, resulting in a total of 224 participants with JIA who underwent the oral health assessment (Fig. 1). For this sub-study, mean age of both participants with JIA and controls was 12.0 years (SD 3.2) (*p* = 0.97). The number of girls among participants with JIA and controls was 133 (59.4%) and 134 (59.8%), respectively. A total of 94.2% (211/224) of all pairs were matched according to mother’s background of origin (an assumption based on family name was made for 10 participants).

Two significant differences between the JIA group and controls were observed (Table 1). First, 137 (64.6%) of mothers in the JIA group versus 153 (73.9%) of mothers in the control group had higher education (*p* = 0.04), while numbers for fathers were 88 (42.1%) and 117 (57.4%) (*p* < 0.01). Second, individuals with JIA reported more frequent gingival bleeding during toothbrushing (57.3% vs. 46.6%, *p* = 0.03). No other significant differences were found between the two groups in sociodemographic or behavioral characteristics (Tables 1, 2). Five participants did not have an initial caries examination (due to temporary misinterpretation of study instructions at the very beginning of data collection), so the subsequent analyses include 219 individuals with JIA and 224 controls. The remaining oral health variables will be presented in an upcoming article.

**Table 1:** Sociodemographic and behavioral characteristics of 224 individuals with juvenile idiopathic arthritis and 224 controls, aged 4–16 years

Variable	Individuals with JIA (n = 224)	Control group (n = 224)	*p* value
Educational level of caregivers, n (%)			
Mother			
High school/vocational school	75 (35.4)	54 (26.1)	0.04
University/college	137 (64.6)	153 (73.9)	
Father			
High school/vocational school	121 (57.9)	87 (42.6)	< 0.01
University/college	88 (42.1)	117 (57.4)	
Share household with, n (%)			
Two caregivers in the household*	172 (79.6)	187 (84.2)	0.21
Only one caregiver in the household	44 (20.4)	35 (15.8)	
Frequency of toothbrushing, n (%)			
Once a day or less/do not know	51 (23.6)	49 (22.2)	0.72
Twice a day, or more	165 (76.4)	172 (77.8)	
Frequency of tooth flossing during the last 3 months, n (%)			
Daily or more	19 (8.8)	19 (8.6)	0.94
Several times weekly or less/do not know	196 (91.2)	201 (91.4)	
Toothpaste, n (%)			
Fluoride toothpaste	204 (94.4)	212 (96.4)	0.34
Fluoride-free toothpaste /do not know	12 (5.6)	8 (3.6)	
During toothbrushing, gingival bleeding occurs, n (%)			
Sometimes or more/do not know	122 (57.3)	103 (46.6)	0.03
Never	91 (42.7)	118 (53.4)	
During toothbrushing, pain or discomfort occurs, n (%)			
Yes/do not know	25 (11.7)	22 (10.0)	0.55
No	188 (88.3)	199 (90.0)	

^*^Also includes living across two households, given two caregivers in both households. Some participants did not respond to the questionnaires. Chi-squared test level of significance *p* < 0.05

**Table 2:** Behavioral characteristics of 98 individuals with juvenile idiopathic arthritis and 98 controls < 12 years

Variable	Individuals with JIA (n = 98)	Control group (n = 98)	*p* value
Age at start of toothbrushing, n (%)			
1 year and older/do not know	81 (83.5)	81 (82.7)	0.87
Under the age of 1 year	16 (16.5)	17 (17.3)	
Does the child get assistance if tooth flossing is performed? n (%)			
Yes	35 (47.3)	39 (51.3)	
No/do not know	39 (52.7)	37 (48.7)	0.62
Cordial/milk on bottle after the age of 1 year, n (%)			
Yes/do not know	45 (46.4)	41 (41.8)	0.52
No	52 (53.6)	57 (58.2)	
Drinks or food offered/available in bed during evening/nights, n (%)			
Yes/do not know	11 (11.5)	9 (9.2)	0.60
No	85 (88.5)	89 (90.8)	

Some participants did not respond to the questionnaires. Chi-squared test level of significance *p* < 0.05

### Concomitant diagnoses and medication use

Concomitant diagnoses and use of medication that were a potential oral health threat to individuals with JIA and the controls, are presented in Additional file 2, Table S1.

### Calibration

Four caries calibration exercises are described in Additional file 5, and the weighted Cohen’s kappa values of these exercises were 0.61, 0.61, 0.91 and 0.65, respectively.

### Caries prevalence at individual level

**Fig. 2:**
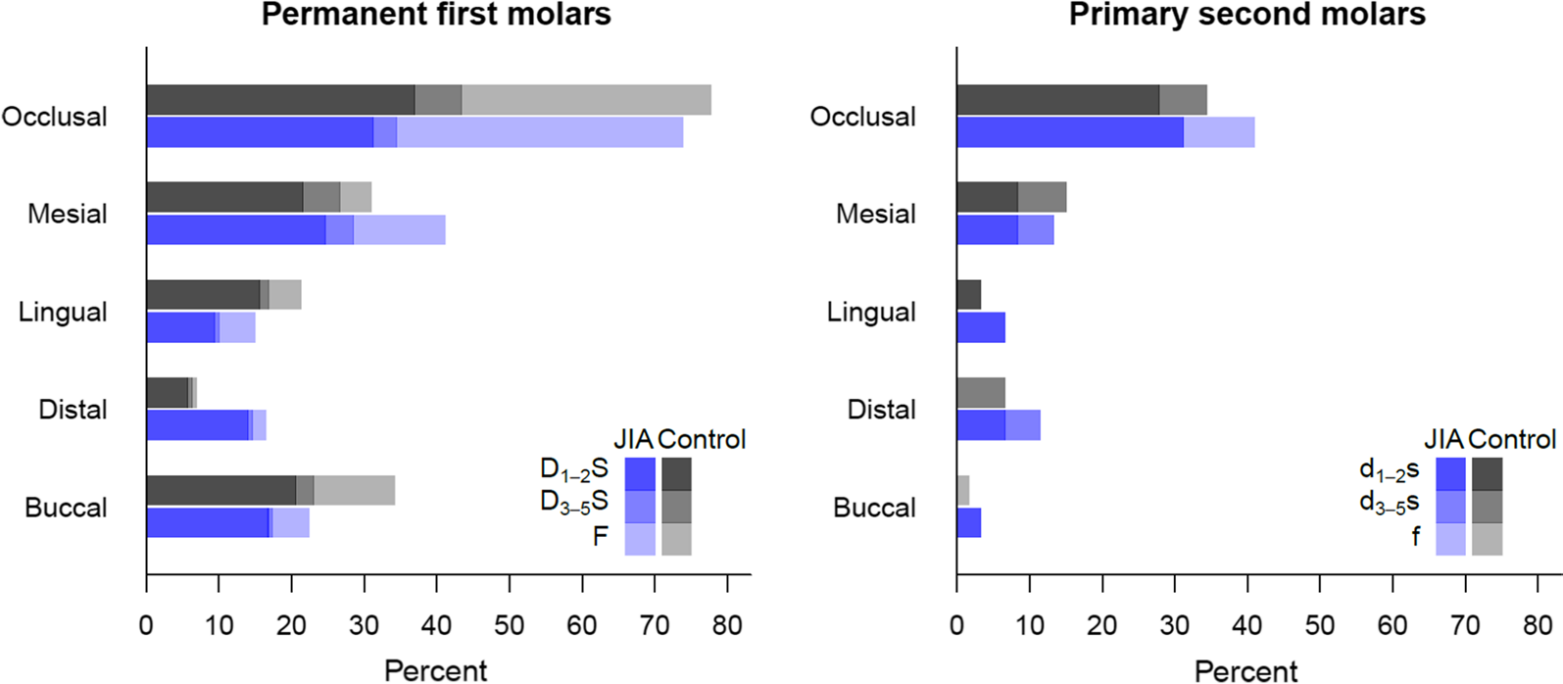
Proportional distributions of d_1-5_fs and D_1-5_FS indices according to tooth surface distribution in children and adolescents with juvenile idiopathic arthritis (JIA) and controls

No significant differences in caries prevalence could be found between the JIA group and the control group regarding primary and permanent molars, whether enamel caries was included or not. In the JIA group, 23.0% (14/61) experienced caries at d_1-5_f-level (decayed and/or filled teeth in the primary dentition, enamel caries included) and 6.6% (4/61) at d_3-5_f-level (enamel caries not included) in one or more primary second molars, while the corresponding proportions in the control group were 27.4% (17/62) and 11.3% (7/62). Individuals in the JIA group and the control group experienced caries in permanent first molars at D_1-5_F-level (decayed and/or filled teeth in the permanent dentition, enamel caries included), 51.9% (82/158) and 50.0% (81/162) respectively. At D_3-5_F-level (enamel caries not included) the corresponding numbers were 36.1% (57/158) and 28.4% (46/162). No permanent molars were extracted or indicated for extraction due to caries, but 2 individuals with JIA and 1 of the control had one or two primary molars extracted (teeth n = 5). In both groups, and for both primary and permanent molars, the occlusal surface was the one most prone to caries, and the proportion of enamel lesions exceeded dentin lesions (Fig. 2).

### Multilevel analyses

Due to the weak correlation between the matching pairs (ICC = 0.08), the original matching in the data was not accounted for in the final multilevel models. Multilevel analysis regressing caries prevalence on JIA status while adjusting for socio-behavioral characteristics and clinical features (intra-oral characteristics) revealed no statistically significant association between JIA status and presence of caries in primary and permanent teeth (d_1-5_ fs/D_1-5_FS > 0, Table 3). Participants with mothers that had high school (or equivalent) education had statistically significantly higher odds of caries (OR = 2.07, 95% CI: 1.24–3.46) than participants with mothers with university-level education. Compared to buccal surface, the occlusal and mesial surfaces of the molars had statistically significantly higher odds of caries, primary and permanent teeth combined. The corresponding odds ratios were 5.06 (95% CI: 3.76–6.83) and 1.66 (95% CI: 1.21–2.29). Compared to the buccal surface, the distal and lingual surfaces had statistically significant lower odds of caries (OR = 0.40, 95% CI: 0.27–0.61 and OR = 0.63, 95% CI: 0.43–0.91, respectively). For a multilevel model with no covariates, the ICC was 0.52, indicating that 52% of the variance in the caries outcome variable was between rather than within individuals. In the adjusted analysis, the ICC was 0.56. The ICCs were found to be statistically significant (*p* < 0.01).

**Table 3:** Multilevel mixed-effects logistic regression analyzing the relationships among health status, sociodemographic and behavioral characteristics, and oral variables with the dichotomized outcome variable presence of caries (d_1-5_ fs/D_1-5_FS > 0 or = 0)

	Unadjusted analysis		Adjusted analysis	
	OR (95% CI)	*p* value	OR (95% CI)	*p* value
Health status				
Control group	1	Ref	1	Ref
JIA group	1.07 (0.70–1.65)	0.77	1.02 (0.62–1.66)	0.95
Educational level of mother				
University/college	1	Ref	1	Ref
High school/vocational school	1.93 (1.20–3.09)	0.01	2.07 (1.24–3.46)	0.01
Educational level of father				
University/college	1	Ref		
High school/vocational school	1.38 (0.87–2.19)	0.17		
Household structure				
Two caregivers*	1	Ref		
One caregiver	1.36 (0.78–2.37)	0.29		
Toothpaste				
Fluoride toothpaste	1	Ref		
Fluoride-free toothpaste**	1.11 (0.40–3.08)	0.84		
Frequency of toothbrushing				
Twice a day or more	1	Ref		
Once a day or less **	1.35 (0.81–2.25)	0.26		
Frequency of tooth flossing during the last 3 months				
Daily or more	1	Ref		
Several times weekly or less**	0.98 (0.44–2.18)	0.96		
During toothbrushing, gingival bleeding occurs				
Never	1	Ref	1	Ref
Sometimes or more **	1.69 (1.09–2.64)	0.02	1.52 (0.94–2.48)	0.09
During toothbrushing, pain or discomfort occurs				
No	1	Ref		
Yes**	3.11 (1.62–5.98)	< 0.01		
Jaw				
Mandible	1	Ref		
Maxilla	1.01 (0.84–1.20)	0.96		
Side				
Right side	1	Ref		
Left side	1.01 (0.84–1.21)	0.91		
Surface				
A: Buccal	1^BDE^	< 0.01*	1^BDE^	< 0.01***
B: Distal	0.45 (0.31–0.65)^ADE^	< 0.01	0.40 (0.27–0.61)^ADE^	< 0.01
C: Lingual	0.66 (0.46–0.93)^DE^	0.02	0.63 (0.43–0.91)^DE^	0.01
D: Mesial	1.67 (1.23–2.26)^ABCE^	< 0.01	1.66 (1.21–2.29)^ABCE^	< 0.01
E: Occlusal	4.86 (3.65–6.45)^ABCD^	< 0.01	5.06 (3.76–6.83)^ABCD^	< 0.01
ICC	0.52		0.56	

JIA, juvenile idiopathic arthritis; ICC, intra-class correlation coefficient

^*^Also includes living across two households with two caregivers in each household. **The answer option “Do not know” is assigned to the variable associated with negative impact on oral health. *Overall *p* value. Surfaces statistically significant in post-hoc analyses are marked with superscript letters. Scheffe adjusted *p* values in post-hoc analyses *p* < 0.05

### Surface-specific caries according to JIA group and dentition (permanent/primary molars)

Participants in both the JIA group and the control group were more likely to have caries on the occlusal surfaces than on the buccal surfaces in both primary and permanent teeth (Table 4). Participants in the JIA group were more likely to have caries on the mesial surfaces than on the buccal surfaces of their permanent teeth, whereas controls were more likely to have caries on the mesial surface than on the buccal surfaces of their primary teeth. All ICCs were statistically significant (all *p* < 0.01). There was a statistically significant interaction between surface in the permanent dentition and group affiliation (JIA/control group) (*p* < 0.01) on presence of caries, whereas in the primary dentition the corresponding interaction was nonsignificant (*p* = 0.66).

**Table 4:** Multilevel mixed-effects logistic regression for surfaces associated with presence of caries (d_1-5_fs/D_1-5_FS > 0 or = 0)

	Individuals with JIA				Control group			
	Permanent dentition		Primary dentition		Permanent dentition		Primary dentition	
	OR (95% CI), Unadjusted	*p* value	OR (95% CI), Unadjusted	*p* value	OR (95% CI), Unadjusted	*p* value	OR (95% CI), Unadjusted	*p* value
A:Buccal	1^DE^	< 0.01*	1^E^	0.01*	1^BE^	< 0.01*	1^E^	0.01*
B:Distal	0.66 (0.38–1.15)^DE^	0.14	4.36 (0.81–23.34)^E^	0.09	0.15 (0.08–0.30)^ACDE^	< 0.01	4.34 (0.46–40.94)^E^	0.20
C:Lingual	0.67 (0.38–1.16)^DE^	0.15	2.18 (0.36–13.14)^E^	0.39	0.56 (0.34–0.91)^BE^	0.02	2.06 (0.18–23.88)^E^	0.56
D:Mesial	2.28 (1.43–3.65)^ABCE^	< 0.01	5.21 (0.99–27.40)^E^	0.05	0.92 (0.59–1.45)^BE^	0.73	14.26 (1.73–117.18)	0.01
E:Occlusal	5.01 (3.20–7.85)^ABCD^	< 0.01	34.25 (6.88–170.54)^ABCD^	< 0.01	3.25 (2.17–4.86)^ABCD^	< 0.01	34.66 (4.34–276.53)	< 0.01
ICC	0.49		0.78		0.54		0.57	

JIA, juvenile idiopathic arthritis; ICC, intra-class correlation coefficient

^*^Overall *p* value. Surfaces statistically significant in post-hoc analyses are marked with the superscript letters of the pertinent surfaces. Scheffe adjusted *p* values in post-hoc analysis *p* < 0.05

### Disease-specific variables associated with caries

No statistically significant associations were found between JIA-specific features and caries in the primary and permanent teeth combined (Table 5). However, analyzing the dentitions separately, the category Oligoarthritis extended (OR = 20.82, 95% CI: 1.57–275.42) and the category Polyarthritis Rheumatoid Factor (RF) negative (OR = 2.48, 95% CI: 1.13–5.45) had statistically significant higher odds for caries, with reference to Oligoarthritis persistent in the primary and permanent dentition, respectively (results not shown in Table 5). In the primary dentition, individuals with MDgloVAS score > 0 had statistically significant higher odds for caries (OR = 5.78, 95% CI: 1.04–32.28), compared to individuals with MDgloVAS score = 0. At surface level (primary and permanent dentition combined), compared to the buccal surface, the occlusal surface (OR = 6.30, 95% CI: 4.11–9.66) and the mesial surface (OR = 2.44, 95% CI: 1.56–3.82) were statistically significantly associated with caries. In the unadjusted analysis the ICC was 0.47 (*p* < 0.01).

**Table 5:** Unadjusted multilevel mixed-effects logistic regression analyzing the relationship between disease-specific features and oral variables, with the dichotomized outcome variable presence of caries (d_1-5_fs/D_1-5_FS > 0 or = 0) in children and adolescents with juvenile idiopathic arthritis (JIA)

	Respondents, n	OR (95% CI)	*p* value
JIA category			
Oligoarthritis persistent	77	1	0.29*
Systemic arthritis	7	0.93 (0.17–5.17)	0.94
Oligoarthritis extended	20	1.88 (0.65–5.43)	0.24
Polyarthritis, RF positive	4	3.07 (0.38–24.83)	0.29
Polyarthritis, RF negative	49	2.19 (0.99–4.85)	0.05
Psoriatic arthritis	8	2.99 (0.67–13.34)	0.15
Enthesitis-related arthritis	23	0.64 (0.21–1.96)	0.44
Undifferentiated arthritis	31	0.84 (0.31–2.26)	0.74
Steroids, ongoing			
No steroids ongoing	215	1	Ref
Steroids ongoing	4	2.31 (0.26–20.58)	0.45
Steroids, ever used			
No steroids ever used	172	1	Ref
Steroids ever used	47	1.30 (0.63–2.71)	0.48
DMARDs, ongoing			
No sDMARDs nor bDMARDs ongoing	73	1	0.78*
sDMARDs, but no bDMARDs ongoing	60	0.82 (0.37–1.79)	0.61
bDMARDs ongoing (with or without sDMARDs)	86	1.06 (0.52–2.16)	0.88
DMARDs, ever used**			
No sDMARDs nor bDMARDs ever used	51	1	0.61*
sDMARDs, but no bDMARDs ever used	79	1.32 (0.58–3.01)	0.50
bDMARDs ever used (with or without sDMARDs)	89	1.48 (0.66–3.31)	0.34
Age at JIA onset			
≤ 6 years	110	1	Ref
> 6 years	109	1.66 (0.90–3.07)	0.11
Disease duration			
≤ 5 years	117	1	Ref
> 5 years	102	1.35 (0.73–2.48)	0.34
Remission status***			
Inactive disease/remission on/off medication	130	1	ref
Continued activity/flare	89	1.16 (0.63–2.16)	0.63
MDgloVAS			
VAS = 0	140	1	Ref
VAS > 0	79	1.26 (0.67–2.37)	0.48
PRgloVAS****			
VAS = 0	106	1	Ref
VAS > 0	108	1.24 (0.67–2.29)	0.49
CHAQ hygiene item tooth brushing****			
Without any difficulty	203	1	Ref
With some/much difficulty/unable to do/not applicable	11	1.03 (0.26–4.09)	0.96
Jaw			
Mandible		1	Ref
Maxilla		1.02 (0.79–1.32)	0.86
Side			
Right side		1	Ref
Left side		1.14 (0.88–1.46)	0.33
Surface			
A: Buccal		1^DE^	< 0.01*
B: Distal		0.82 (0.49–1.38)^DE^	0.46
C: Lingual		0.74 (0.44–1.26)^DE^	0.27
D: Mesial		2.44 (1.56–3.82)^ABCE^	< 0.01
E: Occlusal		6.30 (4.11–9.66)^ABCD^	< 0.01
ICC		0.47	

RF, Rheumatoid Factor; sDMARDs, synthetic disease-modifying antirheumatic drugs; bDMARDs, biologic disease-modifying antirheumatic drugs; MDgloVAS, Physician's global assessment of disease activity; PRgloVAS, Patient's global assessment of overall wellbeing; CHAQ, Childhood Health Assessment Questionnaire; ICC, intra-class correlation coefficient

^*^Overall *p* value. Surfaces statistically significant in post-hoc analyses are marked with the superscript letters of the pertinent surfaces. Scheffe adjusted *p* values in post-hoc analyses *p* < 0.05. For ‘JIA category’, ‘other medication ongoing and ever used’, and post- hoc analysis showed no differences (all *p* values > 0.05). **Include both previously used and ongoing medication. ***Disease activity according to Wallace and the American College of Rheumatology (ACR) provisional criteria [43, 44]. ****Responses (n = 5) are missing

## Discussion

This matched comparative cross-sectional study did not reveal any overall significant difference in caries prevalence between individuals with JIA and controls, both when enamel caries was included or not. Few individuals experienced extractions and only primary molars were extracted. In both primary and permanent dentitions, the occlusal surface was most prone to caries. A lower educational level of participants’ mothers was associated with presence of caries, independent of group affiliation. There was a stronger trend for increased risk of mesial approximal caries (mesial surfaces compared to buccal surface) in the permanent molars of the JIA group, than in the control group. Few JIA disease-specific variables were associated with presence of caries, but no strong conclusions can be drawn because of low number of participants in some of the categories.

A strength of this study is the application of multilevel analyses of surface-specific measures, facilitating exploration and accounting for the clustered structure of the outcome data. As far as we know, multilevel analysis has previously not been applied in studies examining dental caries among young individuals with JIA. Furthermore, improved statistical management accounting for clustering effects of the data is in demand in dental research [21]. In caries epidemiology, the clustered structure of the data (surfaces clustered within teeth, and teeth clustered within individuals) is often ignored and each surface or tooth is treated as an independent observation. This approach is adequate if the within-cluster correlation or intra-class correlation coefficient (ICC) (i.e. correlation between teeth and surfaces for the same individual) is very small, because then the impact of clustering on the analysis can be disregarded [22]. Contrary, in case of increased ICC, suitable statistical analysis for clustered data must be implemented if the unique measure of teeth and surfaces are to be used. The calculated ICCs in this study were statistically significant, demonstrating presence of caries within the individual to be dependent, hence the application of mixed-effect logistic regressions was crucial. Another strength of this study is the large number of participants compared to other studies focusing on caries among young individuals with JIA. In addition, all disease categories were represented in the JIA cohort. The applied multicenter design, including three out of four existing regional pediatric rheumatology centers in Norway, ensured representativeness of the Norwegian population for this group with JIA.

This study also has some weaknesses. For instance, even with the relatively large sampling, possible non-response bias for participants with JIA should not be ignored [23]. The non-responders were slightly but significantly younger, and there was a slightly lower proportion of girls, these factors might have influenced the data and analyses. A weakness might be that potential confounders such as dietary habits were not analyzed. Also, a weak correlation between the matching pairs, indicating that the background variables sex, age, center site, and background origin (western/non-western) were not sufficient matching variables for the present outcome (d_1-5_fs/D_1-5_FS > 0). This resulted in convergence problems if matching was added to the mixed effects models, hence adjustment for the correlation between the matching pairs were omitted.

In 1985 and 1987, Storhaug et al. [24, 25] reported increased caries experience among pre-school and school-aged children with JIA in Norway. This finding is in accordance with international studies of less recent data reporting a high burden of caries among young individuals with JIA [26, 27]. The data in the articles by Storhaug et al. were collected as part of a cohort of disabled individuals, however, including no control group for comparison. There have been no other Norwegian studies more recent than Storhaug et al. that have considered caries epidemiology among young individuals with JIA. It is worth noting that in the intervening years, there has been a substantial caries decline in the western world [28], which should be kept in mind when comparisons with older studies are performed. The modest number of individuals experiencing extractions in the present study emphasizes this trend of caries decline. If we restrict comparisons to studies from the last decade, our finding of no difference in caries burden between individuals with JIA and controls, is mostly in accordance with the literature on this topic [12, 13, 29, 30]. One study even reported of higher caries experience in primary teeth among the participants without JIA compared to participants with JIA, but these findings were based on a small sample size [31]. A recent study has found high levels of caries experience among adolescents with JIA, but the reference of comparison was epidemiological data from different German populations, not a matched control group [32]. There are other contributing factors that probably explain why caries prevalence in young individuals with JIA has in recent years become comparable with the general population. Difficulties with tooth brushing and flossing (due to limited mouth opening and upper limb dysfunction) have repeatedly been highlighted as risk factors for caries in individuals with JIA. Better overall treatment of JIA and especially more effective drugs [9, 10] have presumably increased temporomandibular and upper limb function, thus enabling some individuals to manage more efficient oral hygiene routines than before. It is also well known that orofacial dysfunction or pain may be associated with disadvantageous diet, such as softer, more refined carbohydrates combined with frequent intake. Furthermore, as mentioned in the introduction, alternative sweeteners and sugar alternatives in pediatric drugs in general [11] may have reduced the risk for future caries development.

Low socio-economic status is a known risk factor for caries development, despite the availability of free public dental services for children and adolescents in the Nordic countries [33, 34]. There is also a strong association between social disadvantage and poor child health [35]. In studies of caries epidemiology published in the last decade, social risk factors in individuals with JIA have gained limited attention. The finding in the present study, that parental educational level was lower for the JIA group than the control group, is in line with a recent study by Grevich et al. [30]. One possible explanation for this difference could be that more university-educated parents were willing to have their child participate as controls, and thus our control matching might be a bit skewed despite a high response rate. A difference between our results and Grevich et al. [35] is that the latter did not find parental educational level to be associated with caries outcome, in contrast to the present study which found an association between presence of caries and educational level achieved by the mother, independent of group affiliation.

Surface-specific caries in the permanent dentition differed significantly according to group affiliation. A trend observed in the permanent molars of the JIA group was an increased risk for mesial approximal caries (mesial surface, compared to buccal surface), in contrast to the control group. Effective prophylactic strategies targeting occlusal or buccal/lingual surfaces (e.g., fissure sealants) are not applicable to approximal lesions, so preventing these lesions demands greater effort from the patient themselves (e.g., by flossing). The importance of preventing interproximal invasive procedures are highlighted in the literature as studies suggest approximal composite restorations to be associated with increased caries in the adjacent surface [36] and future periodontal attachment loss as observed in adults [37]. Special attention should be given to young individuals with JIA aimed at preventing and managing approximal lesions.

To various extent, previous researchers have investigated whether disease-specific variables in rheumatological conditions are associated with dental caries [12, 13, 29, 30, 32, 38, 39]. A recent study by Grevich et al. [30] found no associations between categories of JIA and caries status, and no association between JIA duration and caries. Other studies have found no associations between caries and restricted mouth opening [12, 13, 32], involvement of upper limbs [12, 13, 32], temporomandibular pain [32], or medication used [13]. However, a positive correlation between disease activity and functional disability and DMFT index has been reported [29]. In addition, an older study (2004) reported of higher caries experience among participants with polyarthritis (RF negative) than the control group (n = 13), and that caries was associated with TMJ dysfunction [38]. The discrepancies among studies could be explained by improved health care in JIA in general and more effective drugs in particular [9, 10]. In the present study, participants with oligoarthritis extended JIA had statistically significantly higher odds for caries in the primary dentition, and participants with polyarthritis RF negative JIA had statistically significantly higher odds for caries in the permanent dentition, compared to children with oligoarthritis persistent arthritis. When the pediatric rheumatologist reported disease activity (MDgloVAS score > 0), children had statistically significant higher odds for caries in the primary dentition compared to individuals with no activity reported by the physician (MDgloVAS score = 0). The low number of participants in some of the categories means that no general conclusions can be drawn, but it is reasonable to think that disease-specific outcomes consistent with increased disease activity may be associated with caries.

Individuals who are treated with potent immunosuppressives are at increased risk of systemic complications from dental infections [40], and children with JIA should not have untreated decayed teeth, especially in light of the general risk for dental sepsis in severely caries-affected children [41]. This present study shows that, in both individuals with JIA and without, the proportion of enamel lesions exceeded the proportion of dentine lesions in both dentitions, which has been a common recent trend of caries distribution [42]. Surface-specific caries in the permanent dentition differed significantly according to group affiliation (JIA/control group), with a trend towards increased risk of mesial approximal caries (mesial surface compared to buccal surface) in the permanent molars of the JIA group. Clearly, the potential benefit of promotive and preventive dental strategies should be emphasized for this group.

Even though young individuals with JIA have less caries now than they have had historically, clinical practitioners must not ignore that many children and adolescents with JIA use immunosuppressive treatment, which needs special considerations. These children and adolescents must be spared the additional burden of dental caries, implying optimal maintenance and prophylactic oral care.

## Conclusions

No overall difference in caries prevalence between children and adolescents with JIA and controls was found. For both groups, maternal educational level of high school (as opposed to university) and tooth surface were associated with presence of caries. Surface-specific caries in the permanent dentition differed significantly according to group affiliation (JIA/control group). Associations between JIA disease-specific variables and presence of caries cannot be excluded. Due to predominance of enamel lesions, the potential for preventative dental strategies is considerable. To be able to make an appropriate caries risk assessment for children and adolescents with JIA, and to assess whether disease-specific factors are associated with caries, prospective clinical studies with sufficient sample sizes are needed.

## Supplementary Information

**Additional file 1.** Sample size calculation

**Additional file 2.Table S1.** Concomitant diagnoses and medication use among individuals with JIA and the controls that are a potential oral health threat.

**Additional file 3:Table S1A.** Categories for sociodemographic and behavioral characteristics (4–16 years), as originally coded and as re-coded for analyses. **Table S1B.** Categories for behavioral characteristics (< 12 years), as originally coded and re-coded for analyses. **Table S1C.** Categories for disease-specific features, as originally coded (if obtained) and re-coded for analyses.

**Additional file 4.** Elaboration of the JIA-specific clinical background variables, collected by the pediatric rheumatologists

**Additional file 5.** Calibration

**Additional file 6:Table S1.** Illustration of the levels in the multilevel models, according to the dichotomous caries outcome variable and background variables.

## Data Availability

The datasets used and/or analyzed during the current study are available from the corresponding author on reasonable request.

## Abbreviations

ACR: American College of Rheumatology
bDMARDs: Biologic disease-modifying antirheumatic drugs
BW: Bitewing radiographs
CHAQ: Childhood Health Assessment Questionnaire
CI: Confidence interval
CRP: C-reactive protein
dfs/DFS: Decayed and filled surfaces
dmft/DMFT: Decayed, missing and filled teeth
ESR: Erythrocyte sedimentation rate
ICC: Intra-class correlation coefficient
ILAR: International League of Associations for Rheumatology
JIA: Juvenile idiopathic arthritis
MDgloVAS: Clinics physician’s global assessment of disease activity visual analogue scale
NorJIA: The Norwegian JIA Study (Temporo-mandibular Involvement, Oral Health, Uveitis, Bone Health and Quality of Life in Children with Juvenile Idiopathic Arthritis (JIA))
OR: Odds ratios
PDS: Public Dental Service
PRgloVAS: Patient/parent-reported global assessment of overall wellbeing visual analogue scale
RF: Rheumatoid Factor
RIM: Random intercept logistic models
SD: Standard deviations
sDMARDs: Synthetic disease-modifying antirheumatic drugs
TMJ: Temporomandibular joint
VAS: Visual analogue scale

## Footnotes

1 The Norwegian JIA Study – Temporo-mandibular Involvement, Oral Health, Uveitis, Bone Health and Quality of Life in Children with Juvenile Idiopathic Arthritis (JIA).
