# Supplementary material for: Dental caries in children and adolescents with juvenile idiopathic arthritis and controls: a multilevel analysis
Source: BMC Oral Health. 2021 Aug 25;21:417. doi: 10.1186/s12903-021-01758-y (PMC8390188; doi:10.1186/s12903-021-01758-y)
Supplement: Supplementary file 1 — Additional file 1. Sample size calculation [file 12903_2021_1758_MOESM1_ESM.docx]

**Supplementary information**

**Additional file 1**

*Sample size calculation*

The sample size calculation was based on caries figures among children and adolescents with JIA. For primary dentition, the calculation was based on a study by Welbury et al. (1) that reported a mean dmft (decayed at dentin level) of 1.46 in the JIA group and of 0.56 in controls with corresponding standard deviations (SD) of 2.58 and 0.96. The level of significance was set at 0.05 and the statistical power at 80%. By using the two-sided Student sample *t*-*test*, the proposed sample size was 75 participants in each group. Sample size calculation of the permanent dentition was based on a study by Leksell et al. (2) that reported a caries prevalence (decayed at dentin level) in the first permanent molars of 49% in the JIA group and 27% in the control group. The assumptions of an alpha of 5% and power of 80% gave an estimated sample size of 76 for each group (two-sample comparison of percentages). Altogether, for both the primary and the permanent dentition, the above calculation suggested 182 participants in each group in anticipation of a dropout rate of 20% (missing/cancelled appointments). Because enamel caries was included and applied in multilevel analyses, the power of the statistical analysis would increase compared to analysis on only individual level.

1. Welbury RR, Thomason JM, Fitzgerald JL, Steen IN, Marshall NJ, Foster HE. Increased prevalence of dental caries and poor oral hygiene in juvenile idiopathic arthritis. Rheumatology (Oxford). 2003;42(12):1445-51.

2. Leksell E, Ernberg M, Magnusson B, Hedenberg-Magnusson B. Intraoral condition in children with juvenile idiopathic arthritis compared to controls. Int J Paediatr Dent. 2008;18(6):423-33.
