# Supplementary material for: Dental caries in children and adolescents with juvenile idiopathic arthritis and controls: a multilevel analysis
Source: BMC Oral Health. 2021 Aug 25;21:417. doi: 10.1186/s12903-021-01758-y (PMC8390188; doi:10.1186/s12903-021-01758-y)
Supplement: Supplementary file 2 — Additional file 2.Table S1. Concomitant diagnoses and medication use among individuals with JIA and the controls that are a potential oral health threat. [file 12903_2021_1758_MOESM2_ESM.docx]

**Additional file 2**

Table S1. Concomitant diagnoses and medication use among individuals with JIA and the controls that are a potential oral health threat.

|  | Individuals with JIA, n | Control group, n |
| --- | --- | --- |
| Allergy and/or asthma (on medication) | 13 | 7 |
| Autism spectrum disorder | 0 | 1 |
| Neurodevelopmental disability  (sensory and motor disability) | 1 | 0 |
| Psychiatric conditions (on psychotropic medications)  (Attention Deficit Hyperactivity Disorder (ADHD) and depression) | 3 | 1 |
| Coeliac disease | 5 | 0 |
| Diabetes* | 3 | 0 |
| Down syndrome | 2 | 0 |
| Epilepsy** | 1 | 1 |
| Hypothyroidism*** | 2 | 0 |

*Information was recollected by caregiver or participant. Intermittent medication use is not considered, nor previous diagnosis or medication use. Medications used in the treatment strategy of JIA (e.g., antiemetic, eye droplets) are not considered. *Insulin use. **No use of medication. ***Levothyroxine use.*
