# Supplementary material for: Dental caries in children and adolescents with juvenile idiopathic arthritis and controls: a multilevel analysis
Source: BMC Oral Health. 2021 Aug 25;21:417. doi: 10.1186/s12903-021-01758-y (PMC8390188; doi:10.1186/s12903-021-01758-y)
Supplement: Supplementary file 3 — Additional file 3:Table S1A. Categories for sociodemographic and behavioral characteristics (4–16 years), as originally coded and as re-coded for analyses. Table S1B. Categories for behavioral characteristics (< 12 years), as originally coded and re-coded for analyses. Table S1C. Categories for disease-specific features, as originally coded (if obtained) and re-coded for analyses. [file 12903_2021_1758_MOESM3_ESM.docx]

**Additional file 3**

Table S1A. Categories for sociodemographic and behavioral characteristics (4–16 years), as originally coded and as re-coded for analyses.

| Variables | Categories | Original code | New code |
| --- | --- | --- | --- |
|  |  |  |  |
| Share household with^*^ | Mother and father | 1 | 0 |
|  | Only mother | 2 | 1 |
|  | Only father | 3 | 1 |
|  | Other, specify | 4 | 0/1^*^ |
| Educational level of mother/father | Primary school | 1 | 1 |
|  | High school/vocational school | 2 | 1 |
|  | University/college (≥ 4years) | 3 | 0 |
|  | University/college (≤ 5 years) | 4 | 0 |
|  | Unknown | 90 | [sysmiss] |
|  | Missing | 99 | [sysmiss] |
| Frequency of toothbrushing | Never | 1 | 1 |
|  | Most days | 2 | 1 |
|  | Once a day | 3 | 1 |
|  | Twice a day, or more | 4 | 0 |
|  | Do not know | 90 | 1 |
|  | Missing | 99 | [sysmiss] |
| Frequency of tooth flossing during the last 3 months | Several times daily | 1 | 0 |
|  | Twice a day | 2 | 0 |
|  | Daily | 3 | 1 |
|  | Several times weekly | 4 | 1 |
|  | Several times a month, but not weekly | 5 | 1 |
|  | Seldom | 6 | 1 |
|  | Never | 7 | 1 |
|  | Do not know | 90 | 1 |
|  | Missing | 99 | [sysmiss] |
| Toothpaste, n (%) | Fluoride toothpaste for adults | 1 | 0 |
|  | Fluoride toothpaste for children | 2 | 0 |
|  | Fluoride-free toothpaste | 3 | 1 |
|  | Do not use toothpaste | 4 | 1 |
|  | Do not know | 90 | 1 |
|  | Missing | 99 | [sysmiss] |
| During toothbrushing, gingival bleeding occurs | Every day | 1 | 1 |
|  | Most days | 2 | 1 |
|  | Once a week | 3 | 1 |
|  | Sometimes | 4 | 1 |
|  | Never | 5 | 0 |
|  | Do not know | 90 | 1 |
|  | Missing | 99 | [sysmiss] |
| The two questions below are merged and form the basis of “During toothbrushing, pain or discomfort occurs” | | | |
| Question given to the participants ≥ 12 years: **“**Do you sometimes experience pain or discomfort during toothbrushing?” | Yes | 1 | 1 |
|  | No | 2 | 0 |
|  | Do not know | 90 | 1 |
|  | Missing | 99 | [sysmiss] |
| Question given to the participants < 12 years: “Impression of child’s experience during toothbrushing?” | Painful | 1 | 1 |
|  | Unpleasant | 2 | 1 |
|  | Okay | 3 | 0 |
|  | Do not know | 90 | 1 |
|  | Missing | 99 | [sysmiss] |

**The variable “Share household with” was transformed into “Two caregivers in the household” (0), which also includes living across two households with two caregivers in each households, or ”Only one caregiver in the household” (1). The answer “Other, specify” was evaluated and recoded accordingly. [sysmiss] = system missing value.*

Table S1B. Categories for behavioral characteristics (< 12 years), as originally coded and re-coded for analyses.

| Variables | Categories | Original codes | New codes |
| --- | --- | --- | --- |
| Age at start of toothbrushing | Not started | 1 | 1 |
|  | Under the age of 1 years | 2 | 0 |
|  | 1-2 years | 3 | 1 |
|  | Over 2 years | 4 | 1 |
|  | Do not know | 90 | 1 |
|  | Missing | 99 | [sysmiss] |
| Does the child get assistance if tooth flossing is performed? | Yes | 1 | 0 |
|  | No | 2 | 1 |
|  | Do not know | 90 | 1 |
|  | Missing | 99 | [sysmiss] |
| Cordial/milk in bottle after the age of 1 year | Yes | 1 | 1 |
|  | No | 2 | 0 |
|  | Do not know | 90 | 1 |
|  | Missing | 99 | [sysmiss] |
| Drinks or food offered/available in bed during evening/nights | Yes | 1 | 1 |
|  | No | 2 | 0 |
|  | Do not know | 90 | 1 |
|  | Missing | 99 | [sysmiss] |

*[sysmiss] = system missing value*

Table S1C. Categories for disease-specific features, as originally coded (if obtained) and re-coded for analyses.

| Variables | Categories | Original code | New code |
| --- | --- | --- | --- |
| JIA category | Systemic arthritis |  | 1 |
|  | Oligoarthritis persistent |  | 2 |
|  | Oligoarthritis extended |  | 3 |
|  | Polyarthritis, RF positive |  | 4 |
|  | Polyarthritis, RF negative |  | 5 |
|  | Psoriatic arthritis |  | 6 |
|  | Enthesitis-related arthritis |  | 7 |
|  | Undifferentiated  arthritis |  | 8 |
| Age at JIA onset | 6 years or less |  | 0 |
|  | Over 6 years |  | 1 |
| Duration of the JIA disease | 5 years or less |  | 0 |
|  | Over 5 years |  | 1 |
| Disease status on the day of visit* | Continued activity since onset | 1 | 1 |
|  | Flare | 2 | 1 |
|  | Inactive disease on off medication but not yet remission | 3 | 0 |
|  | Remission on medication | 4 | 0 |
|  | Remission off medication | 5 | 0 |
| MDgloVAS | VAS score 0 |  | 0 |
|  | VAS score > 0 |  | 1 |
|  | Missing |  | Missing |
| PRgloVAS | VAS score 0 |  | 0 |
|  | VAS score > 0 |  | 1 |
|  | Missing |  | Missing |
| Hygiene item of CHAQ; toothbrushing | Without any difficulty | 1 | 0 |
|  | With some difficulty | 2 | 1 |
|  | With much difficulty | 3 | 1 |
|  | Unable to do | 4 | 1 |
|  | Not applicable | 90 | 1 |
|  | Missing | 99 | [sysmiss] |
| Medication | | | |
| Steroids ongoing | Steroids, ongoing |  | 0 |
|  | No steroids, ongoing |  | 1 |
| Steroids ever used | Steroids ever used |  | 1 |
|  | No steroids ever used |  | 0 |
| sDMARDs and bDMARDs ongoing | No sDMARDs nor bDMARDs ongoing |  | 0 |
|  | No bDMARDs, but ongoing use of sDMARDs |  | 1 |
|  | No sDMARDs, but ongoing use of bDMARDs. Or bDMARDs use and sDMARDs use ongoing |  | 2 |
| sDMARDs and biologics ever used | No sDMARDs nor bDMARDs ever used |  | 0 |
|  | No bDMARDs ever used, but sDMARDs have been used |  | 1 |
|  | No sDMARDs ever used, but bDMARDs have been used. Or bDMARDs and sDMARDs have been used |  | 2 |

**Disease activity according to Wallace and the American College of Rheumatology (ACR) provisional criteria (1, 2). RF = Rheumatoid Factor.* *sDMARDs = synthetic disease-modifying antirheumatic drugs. bDMARDs = biologic disease-modifying antirheumatic drugs. MDgloVAS = Physician's global assessment of disease activity. PRgloVAS = Patient's global assessment of overall wellbeing. CHAQ = Childhood Health Assessment Questionnaire. [sysmiss] = system missing value.*

1. Wallace CA, Ruperto N, Giannini E, Childhood A, Rheumatology Research A, Pediatric Rheumatology International Trials O, et al. Preliminary criteria for clinical remission for select categories of juvenile idiopathic arthritis. J Rheumatol. 2004;31(11):2290-4.

2. Wallace CA, Giannini EH, Huang B, Itert L, Ruperto N, Childhood Arthritis Rheumatology Research A, et al. American College of Rheumatology provisional criteria for defining clinical inactive disease in select categories of juvenile idiopathic arthritis. Arthritis Care Res (Hoboken). 2011;63(7):929-36.
