# Supplementary material for: Dental caries in children and adolescents with juvenile idiopathic arthritis and controls: a multilevel analysis
Source: BMC Oral Health. 2021 Aug 25;21:417. doi: 10.1186/s12903-021-01758-y (PMC8390188; doi:10.1186/s12903-021-01758-y)
Supplement: Supplementary file 4 — Additional file 4. Elaboration of the JIA-specific clinical background variables, collected by the pediatric rheumatologists [file 12903_2021_1758_MOESM4_ESM.docx]

**Additional file 4**

*Elaboration of the JIA-specific clinical background variables, collected by the pediatric rheumatologists*

Background characteristics in this sub-study were age at onset of JIA, disease duration and JIA category according to the ILAR criteria (1). Disease status on the day of visit was recorded according to Wallace et al. (2) and the American College of Rheumatology (ACR) provisional criteria (3), where inactive disease (on or off medication) included no active arthritis, no fever, no generalized lymphadenopathy, no splenomegaly, no serositis, no rash as a result of JIA, no active uveitis, normal levels of erythrocyte sedimentation rate (ESR) and C-reactive protein (CRP), no morning stiffness exceeding 15 minutes, and MDgloVAS = 0. Clinical remission on medication was defined as six continuous months of inactive disease on medications, and remission off medication as twelve continuous months of inactive disease and no anti-rheumatic medication (2). Previous and on-going medication was registered and categorized into the following groups: 1) no synthetic disease modifying drugs (sDMARDs) nor biologic DMARDs (bDMARDs), 2) sDMARDs, but no bDMARDs, and 3) bDMARDs (with or without sDMARDs). Each group was mutually exclusive. The JIA cohort was also categorized into groups using or not using systemic steroids. All groups were registered according to ongoing medication, or medication ever used, the last included both previously used and ongoing medication. Self-reported physical disability was measured by the validated patient/parent-reported disease-specific childhood health assessment questionnaire (CHAQ), including the hygiene item Tooth brushing (0 = no difficulty and 3 = unable to perform), and the patient/parent overall well-being, PRgloVAS (0 = best and 10 = worst) (4, 5). Global disease activity was registered by the physician using MDgloVAS (0 = no activity and 10 = maximum activity). Both PRgloVAS and MDgloVAS were registered on a 21-point VAS.

1. Petty RE, Southwood TR, Manners P, Baum J, Glass DN, Goldenberg J, et al. International League of Associations for Rheumatology classification of juvenile idiopathic arthritis: second revision, Edmonton, 2001. J Rheumatol. 2004;31(2):390-2.

2. Wallace CA, Ruperto N, Giannini E, Childhood A, Rheumatology Research A, Pediatric Rheumatology International Trials O, et al. Preliminary criteria for clinical remission for select categories of juvenile idiopathic arthritis. J Rheumatol. 2004;31(11):2290-4.

3. Wallace CA, Giannini EH, Huang B, Itert L, Ruperto N, Childhood Arthritis Rheumatology Research A, et al. American College of Rheumatology provisional criteria for defining clinical inactive disease in select categories of juvenile idiopathic arthritis. Arthritis Care Res (Hoboken). 2011;63(7):929-36.

4. Ruperto N, Ravelli A, Pistorio A, Malattia C, Cavuto S, Gado-West L, et al. Cross-cultural adaptation and psychometric evaluation of the Childhood Health Assessment Questionnaire (CHAQ) and the Child Health Questionnaire (CHQ) in 32 countries. Review of the general methodology. Clin Exp Rheumatol. 2001;19(4 Suppl 23):S1-9.

5. Selvaag AM, Ruperto N, Asplin L, Rygg M, Landgraf JM, Forre O, et al. The Norwegian version of the Childhood Health Assessment Questionnaire (CHAQ) and the Child Health Questionnaire (CHQ). Clin Exp Rheumatol. 2001;19(4 Suppl 23):S116-20.
