# Supplementary material for: Dental caries in children and adolescents with juvenile idiopathic arthritis and controls: a multilevel analysis
Source: BMC Oral Health. 2021 Aug 25;21:417. doi: 10.1186/s12903-021-01758-y (PMC8390188; doi:10.1186/s12903-021-01758-y)
Supplement: Supplementary file 5 — Additional file 5. Calibration [file 12903_2021_1758_MOESM5_ESM.docx]

**Additional file 5**

*Calibration*

A plastic-coated instruction sheet of the written description of the five-grade caries diagnosis system with associated photographs (1) was delivered to all examiners. During an initial theoretical session, gradings of available bitewing radiographs and clinical pictures of tooth surfaces were discussed and feedback was given by an expert.

The examiners then evaluated radiographs and pictures of caries and compared their findings with “the expert reference”. In total, four sessions of caries calibration exercises were done prior to and during the study period (Test Caries 1, 2, 3, 4). Most data for assessing caries reliability were based on 71 schoolchildren presenting both primary and permanent teeth. Bitewing radiographs (n = 21) of both primary and permanent teeth and surfaces of extracted teeth (n = 9) were used. For Test Caries 1, only one examiner was included, and this examiner was compared to “an expert reference”. Test Caries 2 was based on the inter-examiner examiner evaluation. Test Caries 3 was an intra-examiner assessment of each examiner with an interval between, while Test Caries 4 was based on comparison to “an expert reference”.

1. Amarante E, Raadal M, Espelid I. Impact of diagnostic criteria on the prevalence of dental caries in Norwegian children aged 5, 12 and 18 years. Community Dent Oral Epidemiol. 1998;26(2):87-94.
