# Supplementary material for: Dental caries in children and adolescents with juvenile idiopathic arthritis and controls: a multilevel analysis
Source: BMC Oral Health. 2021 Aug 25;21:417. doi: 10.1186/s12903-021-01758-y (PMC8390188; doi:10.1186/s12903-021-01758-y)
Supplement: Supplementary file 6 — Additional file 6:Table S1. Illustration of the levels in the multilevel models, according to the dichotomous caries outcome variable and background variables. [file 12903_2021_1758_MOESM6_ESM.docx]

**Additional file 6**

Table S1. Illustration of the levels in the multilevel models, according to the dichotomous caries outcome variable and background variables.

|  |  | **Level** | | |
| --- | --- | --- | --- | --- |
|  |  | Surfaces (I) | Tooth (II) | Individual (III) |
| **Outcome variable** | Presence of caries or no presence (d_1-5_fs/D_1-5_FS > 0 or = 0) * | √ |  |  |
| **Background variables** | Sociodemographic and behavioral characteristics |  |  | √ |
|  | Oral variables** | √ | √ | √ |
|  | Disease-specific variables (JIA) |  |  | √ |

**Presence of caries in the primary or permanent dentition assessed at different thresholds (enamel caries (1-2), dentin caries (3-5)), and filled surfaces). **Jaw (mandible, maxilla), side (right, left), surface (buccal, distal, lingual, mesial, occlusal).*
